# Vitamin D and Infectious Diseases: Simple Bystander or Contributing Factor?

**DOI:** 10.3390/nu9070651

**Published:** 2017-06-24

**Authors:** Pedro Henrique França Gois, Daniela Ferreira, Simon Olenski, Antonio Carlos Seguro

**Affiliations:** 1 Laboratory of Medical Research-LIM12, Nephrology Department, University of São Paulo School of Medicine, São Paulo CEP 01246-903, Brazil; danferreira61@usp.br (D.F.); trulu@usp.br (A.C.S.); 2 Nephrology Department, Royal Brisbane and Women’s Hospital, Herston QLD 4029, Australia; Simon.olenski@health.qld.gov.au

**Keywords:** vitamin D, vitamin D deficiency, infectious diseases, HIV/AIDS, tuberculosis, sepsis, fungal infections, oxidative stress

## Abstract

Vitamin D (VD) is a fat-soluble steroid essential for life in higher animals. It is technically a pro-hormone present in few food types and produced endogenously in the skin by a photochemical reaction. In recent decades, several studies have suggested that VD contributes to diverse processes extending far beyond mineral homeostasis. The machinery for VD production and its receptor have been reported in multiple tissues, where they have a pivotal role in modulating the immune system. Similarly, vitamin D deficiency (VDD) has been in the spotlight as a major global public healthcare burden. VDD is highly prevalent throughout different regions of the world, including tropical and subtropical countries. Moreover, VDD may affect host immunity leading to an increased incidence and severity of several infectious diseases. In this review, we discuss new insights on VD physiology as well as the relationship between VD status and various infectious diseases such as tuberculosis, respiratory tract infections, human immunodeficiency virus, fungal infections and sepsis. Finally, we critically review the latest evidence on VD monitoring and supplementation in the setting of infectious diseases.

## 1. Introduction

Vitamin D (VD) is a fat-soluble steroid essential for life in higher animals. VD is primarily produced in the skin by the direct action of ultraviolet (UV) sunlight, with a much smaller proportion coming from dietary intake [1]. The two major forms of VD are ergocalciferol (VD_2_) and cholecalciferol (VD_3_). VD_2_ is most commonly added to foods given the paucity of naturally occurring VD rich foods, whereas VD_3_ is mainly synthesized in the skin but can be also found in animal-based foods [1].

**Figure 1:**
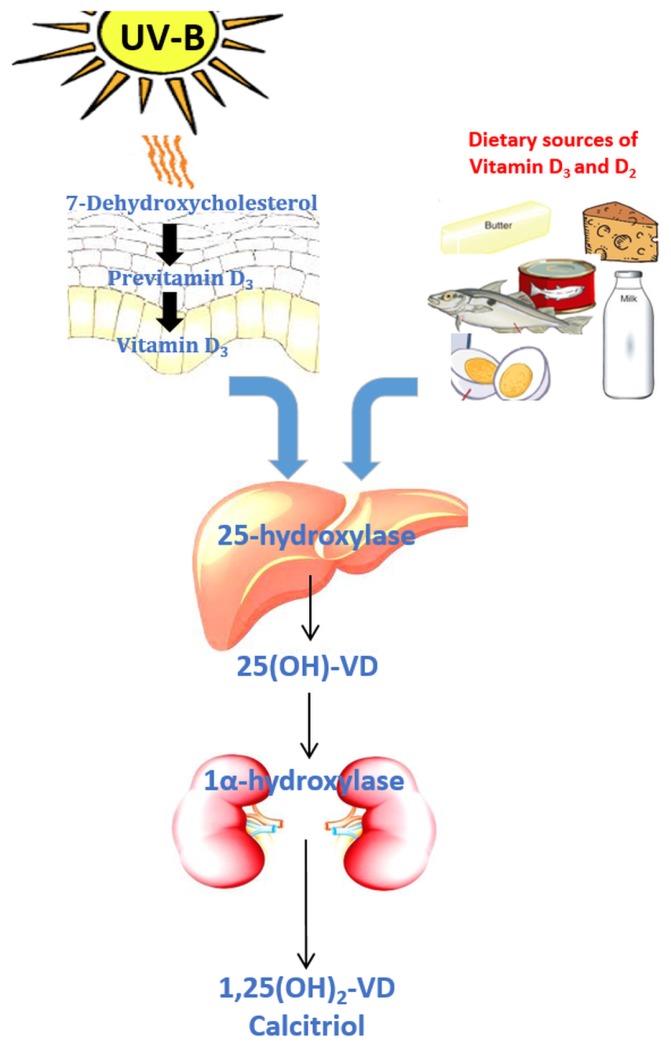
Vitamin D activation and metabolism.

Regarding the VD dermal synthesis pathway, cutaneous-derived 7-dehydroxycholesterol undergoes photolytic conversion by UV sunlight to form previtamin D_3_ [2,3]. Previtamin D_3_ subsequently passes through an immediate non-enzymatic temperature dependent isomerization to form VD_3_ [4]. The pathway of VD conversion in the skin, liver and kidney is illustrated in Figure 1.

Regardless of its source, VD requires a number of steps to become calcitriol (1,25(OH)_2_-VD), the biologically active metabolite of VD [4]. The first step in the bioactivation of VD is its carriage in the serum by VD-binding protein to the liver [4]. VD then undergoes hydroxylation in the liver by 25-hydroxylase (also known as CYP2R1), an enzyme and member of the cytochrome p450 group of enzymes to become 25-hydroxyvitamin D [25(OH)-VD] [2]. 25(OH)-VD is the major circulating form of VD in humans and its plasma levels are routinely measured as a marker of VD status [4].

The final activation step of VD occurs primarily in the kidney, involving a second hydroxylation reaction in which 25(OH)-VD is converted to 1,25(OH)_2_-VD by the enzyme 1α-hydroxylase (also known as CYP27B1) [5]. 1α-hydroxylase is predominantly found in the proximal tubular cells of the kidney but has also been described in other cell types [4].

**Figure 2:**
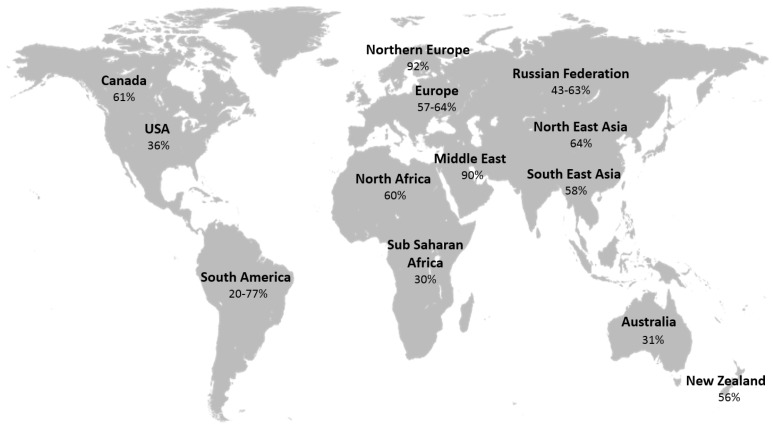
Prevalence of vitamin D deficiency/insufficiency in general population worldwide [7,8,9].

VD sufficiency is defined as having a serum 25(OH)-VD level equal or greater than 75 nmol/L (30 ng/mL) measured at the end of winter or early spring [6]. VD insufficiency is classified as a serum 25(OH)-VD level between 50 and 74 nmol/L (20–29 ng/mL), whereas VD deficiency (VDD) is defined as 25(OH)-VD levels of less than 50 nmol/L (20 ng/mL) [6]. Despite these widely accepted definitions, prevalence studies from around the world have employed different cut-offs for defining VDD, thus making it difficult to obtain a true estimate of this growing public health problem (Figure 2) [7,8,9].

VDD is a major public healthcare burden worldwide with estimates that between 20% and 100% of community-dwelling North American and European elderly present with VDD [10,11]. VDD is typically more prevalent among the elderly due to a combination of reduced UV sunlight exposure, reduced dietary VD intake as well as a reduced ability of the skin to produce VD from diminishing amounts of cutaneous 7-dehydroxycholesterol [12]. Moreover, individuals with darker skin need to be exposed to higher levels of UV radiation to produce sufficient levels of 25(OH)-VD, given the fact that pigmentation reduces the production of VD in the skin [13]. However, in the black population this is not always achieved, especially for those living across most latitudes in North America [13,14]. Furthermore, the prevalence of VDD remains high even in tropical and subtropical countries. A cohort study of 603 healthy Brazilian volunteers from the Clinics Hospital of the University of Sao Paulo showed that after winter the prevalence of VDD was as high as 77.4%, with 26.4% of the study subjects also having secondary hyperparathyroidism [9].

VDD is directly involved in the pathogenesis of rickets, osteoporosis and osteomalacia. Nevertheless, there is a growing body of clinical and laboratory evidence that supports its association with many other vital biological processes, which is also known as the “non-classical” effects of VD [15]. For example, VDD may affect host immunity leading to an increased incidence and severity of various infectious diseases. In this review, we will further discuss the role of VD in modulating the immune system as well as the relationship between VD status and various infectious diseases such as tuberculosis (TB), respiratory tract infections (RTIs), human immunodeficiency virus (HIV), fungal infections and sepsis. Additionally, we will critically review the latest evidence on VD monitoring and supplementation in the setting of infectious diseases.

## 2. Vitamin D and the Immune System

The immune response to infections is a complex and dynamic process involving multiple cell types and soluble factors such as cytokines, chemokines and hormones [16]. Numerous studies support the role of VD in both the innate and adaptive immune responses following viral and bacterial infections [17,18]. In addition, several cells of the immune system express the VD receptor (VDR) and respond directly to 1,25(OH)_2_-VD [19,20].

1,25(OH)_2_-VD has been found to influence cell differentiation within the innate immune system [16]. Previous in vitro studies showed that 1,25(OH)_2_-VD promotes the differentiation of monocytes into macrophages in both mouse and human cells [16]. Lyakh et al. also demonstrated that 1,25(OH)_2_-VD suppresses the differentiation of monocytes into dendritic cells when using in vitro human peripheral blood monocytes exposed to bacterial lipopolysaccharides (LPS) [21]. Furthermore, 1,25(OH)_2_-VD may inhibit the production of interleukin (IL)-12 and IL-10 by LPS-treated monocytes leading to decreased functional capacity of dendritic cells [21]. It is, therefore, proposed that partially mature dendritic cells and low IL-12 levels can induce a tolerogenic state promoting the development of regulatory T-lymphocytes (T cells) with suppressive activity [21,22].

Recent studies have also highlighted the role of VD in enhancing mechanisms of pathogen elimination [15,23]. Apart from dendritic cells, macrophages and their monocyte precursors are the main cells responsible for pathogen phagocytosis and for antigen presentation to T cells [23]. Evidence supporting the concept of an intracrine machinery for VD action within macrophages and monocytes include local activation of VD together with endogenous expression of VDR within these cell types [23]. These findings confirm the study from Koeffler et al. which showed markedly increased 1,25(OH)_2_-VD production after incubating pulmonary alveolar macrophages with interferon gamma (IFN-γ) [24].

**Figure 3:**
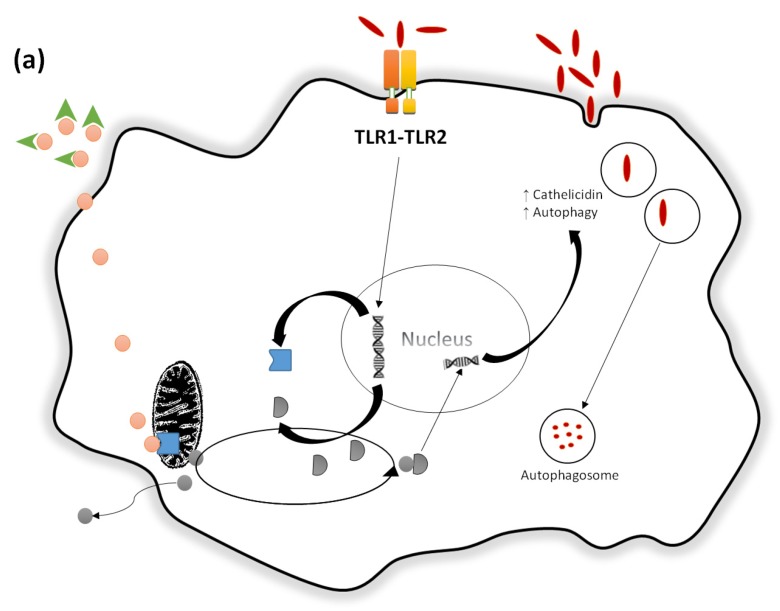

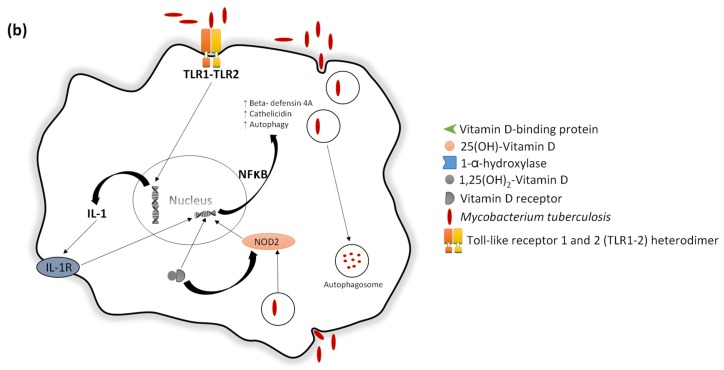
Vitamin D up-regulates the innate defense against *Mycobacterium tuberculosis* (**a**) *M. tuberculosis* binds to the TLR1-TLR2 heterodimer in monocytes leading to the transcriptional induction of VDR and 25(OH)-VD-1-α-hydroxylase; (**b**) Activation of IL-1 and induction of NFκB by TLR1-TLR2. Phagocytosis of *M. tuberculosis* as well as the binding of 1,25(OH)_2_-VD to the VDR activate the intracellular pathogen-recognition receptor NOD2 increasing NFκB activity. In concert, NFκB enhances the expression of cathelicidin and beta-defensin 4A. This ultimately contributes to bacterial killing. Adapted from Hewison, M [15].

Rook et al. also demonstrated that monocytes incubated with VD metabolites have increased anti-tuberculosis activity [25]. In fact, monocytes are able to mediate an immune response against *M. tuberculosis* by phagocytosis or by expressing pathogen-recognition receptors (PRRs) [15]. Among the PRRs, the Toll-like receptors (TLRs) are the most prominent family of receptors triggering anti-microbial activity against *M. tuberculosis* [26]. A heterodimer of TLR1 and TLR2 has been described binding to a *M. tuberculosis* lipoprotein leading to transcriptional induction of VDR and 25(OH)-VD-1-α-hydroxylase [27]. Moreover, activated TLR1-TLR2 heterodimers induce transcription of IL-1 which subsequently increases nuclear factor κB (NFκB) activity via intracrine signaling [15]. Hence, 25(OH)-VD enters the monocyte and after conversion to 1,25(OH)_2_-VD in the mitochondria, binds to the VDR and ultimately acts as a transcription factor for human cathelicidin—an antimicrobial peptide [15,28,29]. It is hypothesized that the transcriptional regulation of cathelicidin by VDR represents an evolutionary change presumably acquired when primates became more exposed to sunlight [23]. Figure 3a,b illustrates VD’s immunomodulatory effects in up-regulating the innate defense against *M. tuberculosis*.

Recent research has shown that regulation of the cathelicidin antimicrobial peptide (CAMP) gene by both the VDR and 1,25(OH)_2_-VD is not evolutionary conserved in mice or rats [29,30]. It is proposed that in rodents the CAMP gene is not controlled by the VD pathway due to the absence of a VD response element (VDRE) in its promoter [29,30]. However, the CAMP promoter was found to be present in humans and primates (especially the great apes) suggesting that this could be a specific adaptation of the immune system after the primates [29,31]. CAMP genes can be activated by both TLR ligands and 1,25(OH)_2_-VD, providing specific mechanisms for humans and primates to modulate their immune response and counteract pathogen-mediated suppression [30,32]. Therefore, the absence of VD regulation in the CAMP gene of mice and rats makes rodents limited models to study VD-signaling and the effects on this antimicrobial peptide [30].

Furthermore, several studies suggest that VD might also have a role in the defense against viral infections [23,33,34]. Hansdottir et al. first demonstrated that in vitro human respiratory epithelial cells expressed higher levels of 1-α-hydroxylase and lower levels of inactivating 24-hydroxylase, thereby increasing overall activation of VD [34]. Similarly, locally activated VD resulted in downstream effects including upregulation of both the cathelicidin peptide gene and TLR co-receptor CD14 [34]. Moreover, 1,25(OH)_2_-VD bound to the VDR induces transcription of an antimicrobial peptide through a VDRE within the promoter region of human gene DEFB4 for defensins β-defensin 2 and 4A [15,33]. Defensins have multiple roles including acting as antibacterial effectors of the innate immune response and also as antiviral peptides [35]. Defensins can also inhibit viral replication and directly induce viral membrane disruption [36].

1,25(OH)_2_-VD’s anti-inflammatory effect in human T cells is partially mediated by NFκB [37]. NFκB is a transcriptional factor composed of different subunits, e.g., p65 (activating) and p50 (inhibitory), that controls the transcription of inflammatory proteins including cytokines and chemokines [16,37]. Yu et al. illustrated that 1,25(OH)_2_-VD had an inhibitory effect on NF_K_B by reducing its protein expression in both the cytosolic and nuclear compartments of T cells [37]. Moreover, 1,25(OH)_2_-VD has been reported to shift the T helper (Th) cell response from Th1 to Th2 [23]. Multiple in vitro studies revealed that 1,25(OH)_2_-VD inhibited the development of Th1 mediated immunity, which is required for the induction of cellular responses [16,23,30,38,39]. Likewise, Cantorna et al. demonstrated that 1,25(OH)_2_-VD treatment suppressed the Th1 response and protected animals from experimental autoimmunity [40]. Therefore, this T cell shift promoted by 1,25(OH)_2_-VD might act in reducing Th1-mediated tissue damage via inhibition of the Th1 cytokine IFN-γ and an increase in the Th2 cytokines IL-4, IL-5 and IL-10 [23,41]. Overall, while VD can stimulate antibacterial activities in innate immune cells and epithelial cells, VD can simultaneously exert anti-inflammatory effects on adaptive immunity.

Human B cells much like their counterpart T cells also express the VDR [42]. Additionally, B cells also express mRNAs for enzymes involved in VD activity such as 1α-hydroxylase and 24-hydroxylase [41]. Recent studies have suggested that 1,25(OH)_2_-VD may have direct effects on B cell homeostasis by inhibiting its differentiation into plasma cells and modulating the production of immunoglobulins [23,41].

## 3. Vitamin D and Tuberculosis

TB is a multi-systemic bacterial infection caused by Mycobacterium tuberculosis [43]. The latest World Health Organization (WHO) global report estimated that 10.4 million people developed TB in 2015, of which 1.8 million died from the disease [44]. The public health burden is even higher, considering that approximately one-third of the world's population has latent TB [44].

TB is an infectious disease known to have a strong association with poverty [45]. Over 95% of cases and deaths are in developing countries [44]. Several reports have shown a linkage between TB and malnutrition, presumed to be due to multiple factors including an increased catabolic state, a lower food intake as well as from treatment side effects [46,47]. Additionally, malnutrition weakens the immune system thus increasing the risk of TB reactivation [46,47]. Overall, nutrient deficiency enhances susceptibility to TB infection, which in turn aggravates undernutrition, creating a vicious cycle [46].

Micronutrients are essential elements to modulate various body reactions such as cell growth and repair, thereby contributing to homeostasis and disease prevention [48]. Previous studies reported lower levels of micronutrients such as vitamin A, D, E, zinc, iron and selenium in TB-infected individuals [46]. Moreover, there has been an increasing amount of literature showing VDD as an independent risk factor for TB infection [49,50,51,52,53]. In recent years, two systematic reviews with meta-analysis have been undertaken to assess this association [52,53]. Nnoaham et al. found a positive correlation between VDD and active TB, although VDD was not uniformly defined among the included studies [52]. Similarly, Zeng et al. reported in a meta-analysis of 15 studies (including 1440 cases and 2558 controls) that 25(OH)-VD levels below 25 nmol/L significantly increased the risk of active TB, while 25(OH)-VD levels between 26 and 50 nmol/L represented only a trend towards an increased risk [53].

The first attempt to use VD supplementation in treating TB was reported by Williams in 1849 [54,55]. He demonstrated that 234 TB cases had an improvement in their symptoms after a few days of treatment with fish liver oil [55]. It was only in 1950 after the identification of VD’s structure that Charpy first used pharmacologic doses of VD_2_ in the management of cutaneous TB [54].

Over the past decade eight RCTs and one complementary report of an RCT comprising over 1700 patients have been performed aiming to evaluate the effects of adjuvant VD in the setting of pulmonary TB in adults (Table 1) [56,57,58,59,60,61,62,63,64]. Regarding sputum conversion rates, three studies showed significantly higher sputum conversion in patients that received VD supplementation [54,62,63]. In one study, VD treatment was effective only in individuals with a specific genotype of the VDR, i.e., the tt polymorphism [64]. One study included mortality rate as an outcome but did not find any difference between groups (VD supplementation versus placebo) after 12 months of follow up, however, the authors could not detect increased levels of 25(OH)-VD in the intervention group compared to the placebo group [58]. Four out of eight trials included clinical assessments such as TB score in the outcomes, of which only one study showed beneficial effects of VD supplementation [58,59,60,62]. Furthermore, VD was generally well tolerated in patients already receiving TB treatment. Adverse events and serum calcium levels were similar between groups (VD supplementation and placebo) in all the studies. Moreover, the frequency of hypercalcemia reported in the trials reviewed here was surprisingly low. Although not directly related to VD supplementation, three cases of paradoxical upgrading reactions were reported in patients allocated to VD groups [57,60]. Finally, three studies demonstrated beneficial immunomodulatory effects of VD supplementation in TB-infected individuals [60,62,64].

Taken together, results from these studies must be interpreted with caution since they present several concerns and limitations. Firstly, the doses of VD employed as well as the primary outcomes were strikingly different among the studies. Additionally, four studies did not evaluate VD levels after supplementation and one study did not provide VD baseline levels [56,59,60,63]. Lack of VD measurement in some of these studies makes it impossible to demonstrate that individuals in the supplementation group had significantly higher serum VD compared to controls. Therefore, given the low-cost and high safety profile of VD supplementation together with its well-described pleiotropic effects, there is a need for better quality RCTs in TB-infected subjects. Future RCTs should be adequately powered to detect the effects of adjunctive VD and have more robust outcome measures (including clinical, radiological and laboratory data).

## 4. Vitamin D and RTI

Evidence supporting the hypothesis that VDD may predispose to influenza infection derives from observational studies, highlighting the seasonal influence of low sun exposure in both conditions [65,66]. Berry et al. described a linear relationship between VD levels and RTI in a cross-sectional study of 6789 British adults. The authors found that for each 10 nmol/L increase in 25(OH)-VD there was a 7% lower risk of RTI [66]. Accordingly, data from the US Third National Health and Nutrition Examination Survey (NHANES) which included 18,883 adults showed an independent association between serum 25(OH)-VD and recent upper RTI [67]. This correlation was even stronger in individuals with underlying lung disease [67]. Furthermore, similar observations have been reported in other studies, some including children as well as different ethnic groups [68,69,70].

Clinical trials evaluating the effect of VD supplementation in preventing RTIs have reported contrasting protocols and results. Bergman et al. conducted a systematic review with meta-analysis assessing the effect of VD supplementation on the risk of RTI [33]. Pooled data from 11 RCTs comprising 5660 individuals indicated that VD supplementation is safe and might have a beneficial effect in preventing RTIs [33]. More recently, Martineau et al. published a systematic review with meta-analysis of individual participant data from 25 RCTs including 10,933 subjects [71]. Trials were conducted in 14 countries and 19 out of 25 studies assessed baseline 25(OH)-VD levels. All RCTs supplemented VD_3_ orally with different protocols of administration: bolus doses every month to every three months (seven studies using doses ranging from 30,000 to 200,000 IU/month), weekly doses (three studies using doses of 1400, 10,000 and 20,000 IU respectively), daily doses (12 studies using doses ranging from 300 to 4000 IU/day) and a combination of bolus and daily doses (three studies using bolus doses from 96,000 to 120,000 IU and daily doses from 400 to 4000 IU/day). High quality evidence from 25 RCTs with moderate heterogeneity revealed that VD_3_ supplementation reduced the risk of experiencing at least one RTI (number needed to treat (NNT) to prevent one episode of RTI = 33, 95% CI 20–101). Furthermore, subgroup analysis of 15 RCTs indicated that daily or weekly VD_3_ administration protected against RTI (NNT = 20, 95% CI 13–43), while no protective effect was seen in the 10 RCTs that used bolus doses of VD_3_. Moreover, VD_3_ supplementation was effective in preventing RTIs irrespective of baseline 25(OH)-VD levels, albeit the protective effects were greatest among subjects with more pronounced VDD [71].

Taken together, the RCT data currently available suggest that VD_3_ supplementation may represent a novel and safe indication for preventing RTIs [71]. In addition, daily or weekly doses seem to be more efficient than pulse therapy [33,71]. Finally, individuals with lower levels of VD might benefit more from the supplementation [70,71].

## 5. Vitamin D and Human Immunodeficiency Virus Infection

VDD is a common finding in HIV-infected patients. Coelho et al. found that 63 out of 97 adult patients on antiretroviral therapy presented with insufficient levels of VD [72]. The investigators also showed that VD supplementation over 24 weeks improved CD4+ cell count recovery [72]. Furthermore, a study from the US including over 1700 women found that the prevalence of VDD among HIV+ subjects was 60%, of which African American women had the highest rates of VDD of all the ethnic groups [73]. Similarly, a Brazilian cohort study of adolescents and young adults with perinatally acquired HIV revealed that the prevalence of VD insufficiency was 29.2% [74].

In a prospective study of 398 adult patients with Acquired Immune Deficiency Syndrome (AIDS) on highly active antiretroviral therapy (HAART), those with VDD showed less recovery of CD4+ cells after 18 months of HAART [75]. Coelho et al. also reported a high prevalence of VD insufficiency among HIV-infected subjects with a CD4+ T cells nadir < 50 cells/mm^3^ [72]. Hence, the investigators showed a significant positive correlation between CD4+ T cells and 25(OH)-VD levels after 24 weeks of VD supplementation [72]. Therefore, VD supplementation in these patients might be a valuable adjuvant therapy to increase CD4+ cell recovery during HAART.

To date, four cohort studies have investigated the association between VD status and mortality [76,77,78,79]. Haug et al. reported lower serum levels of 1,25(OH)_2_-VD in symptomatic HIV-infected patients when compared to asymptomatic HIV-infected subjects [76]. The investigators also demonstrated that HIV-infected patients with abnormally low 1,25(OH)_2_-VD levels also had shorter survival times, although serum 1,25(OH)_2_-VD was not correlated with 25(OH)-VD levels in this study [76]. Moreover, in a cohort study of 884 HIV-infected pregnant women from Tanzania, those with lower 25(OH)-VD levels had a more rapid progression of HIV infection as well as higher all-cause mortality [77]. Sudfeld et al. also showed that VDD may lead to an increased mortality in those on HAART and this association occurred independently of impaired CD4 T cell reconstitution [78]. Finally, VDD was independently related with higher mortality and higher prevalence of AIDS-defining conditions among HIV-infected subjects followed up in the EuroSIDA study [79].

HIV-infected individuals have lower bone mineral densities and are at higher risk for bone fractures when compared to the general population [80,81,82]. Additionally, a few clinical studies suggested that VD supplementation might reduce bone turnover and increase bone mineral density [83,84]. In the setting of aging HIV patients, where osteopenia and osteoporosis are highly prevalent, assessing and supplementing 25(OH)-VD might be an important adjuvant measure to prevent and treat bone disease [85]. Furthermore, Sudjaritruk et al. demonstrated that VDD was associated with secondary hyperparathyroidism, increased bone turnover and bone loss in perinatally HIV-infected adolescents (aged 10–18 years) on HAART [86]. The authors suggested that VD supplementation might prevent bone loss, particularly if administered before the onset of hyperparathyroidism [86].

Efavirenz, a non-nucleoside reverse transcriptase inhibitor, has also been associated with low bone mineral density [85,87]. In addition, among HIV-infected subjects, those on efavirenz-containing therapy were at a higher risk for VDD [73,88]. The mechanism through which efavirenz lowers VD levels, and therefore may reduce bone mineral density, is through induction of 24-hydroxylase which increases the catabolism of 25(OH)-VD into the inactive form 24,25(OH)_2_-VD [85].

Similarly, tenofovir disoproxil fumarate (TDF) is another antiretroviral agent from the nucleotide reverse transcriptase inhibitor class and is associated with hypophosphatemia secondary to proximal renal tubular dysfunction, hyperparathyroidism and increased bone loss [85]. Moreover, chronic use of TDF may also lead to a decreased glomerular filtration rate [89]. An experimental study from our laboratory using a rat model demonstrated that VDD aggravated TDF nephrotoxicity and induced hypertension and hyperlipidaemia [90].

VDD may lead to worse outcomes in patients with AIDS through a number of different mechanisms including decreasing bone mineral density, altering immunologic state, as well as increasing the risk for adverse renal and cardiovascular adverse events. Although RCTs are needed, we propose that VD levels should be carefully monitored in AIDS patients, particularly for those on TDF-containing HAART. AIDS patients with VDD might benefit from VD supplementation to improve long-term outcomes.

## 6. Vitamin D and Fungal Infections

There has been an increase in the incidence and prevalence of fungal infections worldwide over the past three decades [91]. Fungal infections are one of the major causes of human disease, especially among immunocompromised and hospitalized patients with severe underlying diseases [91,92,93]. There are more than 150 known species of fungi in the world, of which only 15 species have been isolated in patients. 95% of fungal infections are caused by only 5 species: *C. albicans*, *C. glabrata*, *C. parapsilosis*, *C. tropicalis* and *C. krusei* [92,94,95,96]. Furthermore, *Candida* spp. have been reported as the fourth and seventh most common cause of nosocomial bloodstream infection in the US and Europe respectively [92,97]. According to the Centers for Disease Control and Prevention (CDC), the annual incidence of *Candida* infection was reported as 7.28/100,000 inhabitants between 1992 and 1993 [98]. Another surveillance study found much higher incidence rates in the states of Atlanta and Baltimore (USA) between 2008 and 2011, respectively 13.3 and 26.2 per 100,000 inhabitants [99,100].

Fluconazole is the most commonly prescribed antifungal drug employed to treat *Candida* infections [101]. However, prolonged antifungal prophylaxis and treatment have been associated with increased resistance to antifungal agents [102,103]. Reports from various centers also showed that *C. albicans* and *C. tropicalis* became resistant to fluconazole leading to clinical therapeutic failure [102]. Moreover, there are very few resources available regarding the use of antifungal agents in the setting of antifungal failure. Therefore, in the last decade, antifungal resistance became a matter of great public health concern [97,104].

Despite its high toxicity, amphotericin B (AmB) remains the gold standard antifungal agent for the treatment of systemic mycoses, especially due to its greater activity against the fungal ergosterol membrane [105]. Regarding the different AmB formulations, both liposomal AmB and AmB solubilized in lipid emulsion (AmB/LE) seem to be less toxic, albeit the latter represents a lower cost alternative formulation with similar activity [106]. In a recent experimental study, we investigated whether VDD was a risk factor for AmB-induced nephrotoxicity [107]. We showed that VD deficient rats presented with renal dysfunction associated with increased urinary magnesium excretion following treatment with AmB/LE [107]. On the other hand, VD sufficient animals treated with AmB/LE exhibited normal renal function. Therefore, based on this experimental study we suggest that VDD might be a risk factor for AmB-induced nephrotoxicity regardless of the formulation prescribed [107].

In another experimental study, *Candida*-infected mice treated with low-dose 1,25(OH)_2_-VD had reduced fungal burden and better survival when compared with untreated mice [108]. However, those animals treated with higher doses of 1,25(OH)_2_-VD (0.1 and 1 μg/kg) had poorer outcomes [108]. The authors concluded that the beneficial immune response of VD supplementation was only achieved with lower doses of 1,25(OH)_2_-VD (i.e., 0.001 and 0.01 μg/kg) [108]. Furthermore, data from in vitro *C. albicans* stimulation showed that 1,25(OH)_2_-VD induced an anti-inflammatory profile of cytokines as well as mediated inhibition of TLR2 and TLR4 [109]. In the clinical setting, Lim et al. showed that a cohort of 28 patients with *Candida* blood stream infection had lower levels of 25(OH)-VD when compared with 78 hospitalized patients and 30 healthy volunteers [108].

Cryptococcal meningitis (CM) has been described as one of the most important opportunistic infections related to HIV and the leading cause of death in AIDS patients living in low-resources areas [110,111,112,113]. In a South African study, 25(OH)-VD levels were assessed in 150 patients with CM and 150 HIV-infected matched controls [112]. The authors aimed to evaluate the association between VD levels and disease severity, immune response and microbiological clearance [112]. The prevalence of VDD, defined as plasma 25(OH)-VD ≤50 nmol/L, was 74% in this study. Nevertheless, VDD was not associated with microbiological clearance in this study [112]. Additionally, there was no significant association between 25(OH)-VD levels and fungal load or cytokine profile in the cerebrospinal fluid [112].

At present, many questions regarding the role of VDD as a potential risk factor for fungal infections remain unanswered. Further research could explore the mechanisms of VD in modulating the inflammatory response, the association between VDD and fungal infections, as well as whether VD supplementation improves outcomes in those patients with fungal infections.

## 7. Vitamin D and Sepsis

Sepsis is a clinical syndrome that has physiological, pathological and biochemical abnormalities induced by infection [114]. Bacterial infections are the most common cause of sepsis, but virtually any infectious organism (e.g., viruses, fungi, and parasites) can precipitate it [115]. According to the third international consensus definitions for sepsis and septic shock (Sepsis-3), sepsis should be defined as life-threatening organ dysfunction caused by a dysregulated host response to infection [114].

Despite significant advances in modern medicine, sepsis remains a major healthcare burden worldwide [116]. It is both an important cause for hospital admission and the leading reason for admission to the intensive care unit (ICU) [117]. Moreover, sepsis is currently the tenth overall leading cause of death in the United States and the commonest cause of non-cardiac death among critically ill patients in a non-coronary ICU [118].

Recent research has shown an increased incidence and mortality from sepsis during the winter months [117]. While the increase in viral RTIs during winter might be a confounding factor regarding the seasonal variation of sepsis, other elements might also contribute to this epidemiological observation [117]. Specifically, VD has been in the spotlight as a potential contributing factor given that it presents with similar seasonal variability [117,119]. Observational studies have revealed correlations between lower levels of VD with the risk of sepsis and increased mortality [120,121]. In fact, two recent systematic reviews with meta-analysis pooled this data together showing an increased risk of sepsis in subjects with lower VD levels [122,123]. However, there are several limitations regarding this evidence. Firstly, both systematic reviews, which were published within one year of each other, included different studies in the meta-analysis. Secondly, the majority of the studies were retrospective in nature. Finally, the definitions used for sepsis and VDD or VD insufficiency were highly heterogeneous among the included studies. Although lower levels of VD might be associated with a higher risk of sepsis, larger prospective studies are needed to confirm this causal relationship.

There is a relatively small body of literature evaluating VD supplementation in the context of sepsis. Currently, seven RCTs have been published on VD levels in the adult ICU setting, of which only two evaluated the effects of supplementation in those patients with sepsis [124]. Quraishi et al. explored the effects of a single dose of VD_3_ (200,000 or 400,000 IU orally or via naso/oro-gastric tube) versus placebo in a small sample of subjects (*n* = 30 in total) within 24 h of new-onset severe sepsis or septic shock [125]. The authors reported no significant difference in clinical outcomes between the study groups [125]. Similarly, Leaf et al. administered a single dose of 1,25(OH)_2_-VD (two mcg intravenously) to 37 critically ill patients with severe sepsis or septic shock versus placebo (*n* = 31) [126]. Clinical outcome measures were assessed as secondary outcomes, with subjects randomized to 1,25(OH)_2_-VD showing no difference in clinical outcomes (e.g., organ function indexes, length of stay, and mortality) [126]. Additionally, no adverse events related to VD supplementation were reported in the two RCTs available [125,126]. We also identified one ongoing study (ClinicalTrials.gov identifier NCT02684487) of which the results are not yet available.

Further research should be undertaken before introducing VD supplementation into the routine clinical care of sepsis. It is yet to be determined the type of VD metabolite, the appropriate dose and the timing of administration that would improve clinical outcomes in those patients with sepsis. Another challenge might be normalizing VD levels in critically ill patients, as well as maintaining optimum VD levels in the general population, with the aim of preventing sepsis.

## 8. Conclusions

In summary, the studies reviewed here highlight the role of VD beyond being only a simple bystander in various infectious diseases. VDD might in fact contribute to the pathogenesis of several infectious diseases by negatively modulating vital processes such as the innate and adaptive immune response. Currently the strongest available evidence supports the use of daily or weekly VD_3_ supplementation as prophylaxis for acute RTIs, especially in individuals with more severe VDD. This benefit may also extend to subjects at higher risk for RTIs, such as those patients with asthma [71]. For the other infectious diseases reviewed here, the RCT data available do not support prophylactic use of VD. While a number of observational studies have evaluated the relationship between VDD and active TB, RCTs on VD supplementation in adults with active TB reported heterogeneous protocols and contrasting results, thus making it difficult to draw conclusions of which supplementation scheme would most benefit TB-infected subjects. Moreover, there is a paucity of studies exploring the association between VDD and HIV, as well as between VDD and fungal infections, and much more research here is warranted. VD toxicity, also known as hypervitaminosis D, is a potentially life threating condition usually caused by excessive VD supplementation. It may lead to hypercalcemia and subsequent renal and cardiovascular damage. Although VD supplementation was generally safe in the studies reviewed here, long-term supplementation above the upper intake level (maximum daily intake without adverse health effects) can increase the risk of toxicity [127]. While symptoms of toxicity are unlikely at daily intakes below 10,000 IU/day, the Food and Nutrition Board at the Institute of Medicine of the National Academies in the US recommended a VD_3_ dose of 4000 IU as the upper intake level for adults [127,128]. Nevertheless, 25(OH)-VD levels above 125–150 nmol/L should be avoided, as they might be associated with increased cardiovascular events and all-cause mortality [127]. Finally, it remains unclear whether VD supplementation has any benefit for patients with infectious diseases and normal VD levels. A fruitful area for future well-designed RCTs would be to evaluate the role of adjunctive VD therapy in infectious diseases, particularly in those subjects with concomitant VDD.

## Figures and Tables

**Table 1:** Overview of the randomized controlled trials evaluating VD supplementation on Tuberculosis.

Author	Study Design	Number of Patients	Dose of Vitamin D	Adverse Events	Primary Outcomes	Conclusion
Coussens et al. [64] ^#^	Double-blind, randomized, placebo-controlled	95	100,000 IU VD_3_ PO (4 doses)	Not reported	Sputum smear and culture conversion Circulating immune response	Improved both outcomes
Daley et al. [56]	Double-blind, randomized, placebo-controlled	247	100,000 IU VD_3_ PO (4 doses)	Not correlated with intervention	Sputum culture conversion	No difference between groups
Martineau et al. [57]	Double-blind, randomized, placebo-controlled	146	100,000 IU VD_3_ PO (4 doses)	Paradoxical upgrading reaction (*n* = 2)	Sputum culture conversion	Improved outcome only for tt genotype (VDR receptor)
Mily et al. [62]	Double-blind, randomized, placebo-controlled	288	5000 IU/day VD_3_ PO (2 months)	Not correlated with intervention	Sputum culture conversion Clinical symptoms ^¶^	Improved only culture conversion
Nursyam et al. [63] ^±^	Double-blind, randomized, placebo-controlled	67	1000 IU/day VD * PO (2 months)	Not reported	Sputum smear conversion Radiological changes	Improved both outcomes
Ralph et al. [59]	Double-blind, randomized, placebo-controlled	200	50,000 IU VD_3_ PO (2 doses)	Similar between groups	Sputum culture conversion Clinical symptoms/lung function test	No difference between groups
Salahuddin et al. [60]	Double-blind, randomized, placebo-controlled	259	600,000 IU VD_3_ IM (2 doses)	Paradoxical upgrading reaction (*n* = 1)	Weight gain Radiological changes	Improved both outcomes ^&^
Tukvadze et al. [61]	Double-blind, randomized, placebo-controlled	199	50,000 IU 3×/week VD_3_ PO (8 weeks) and 50,000 IU q2week (8 weeks)	Similar between groups	Sputum culture conversion	No difference between groups
Wejse et al. [58]	Double-blind, randomized, placebo-controlled	367	100,000 IU VD_3_ PO (3 doses)	Similar between groups	Clinical symptoms	No difference between groups

VD: Vitamin D; VD_3_: Cholecalciferol; PO: *per os*, oral administration; IM: Intramuscular; VDR: Vitamin D receptor q2week: Every other week; ^#^ Coussens et al. reported complementary data from the RCT conducted by Martineau et al.; ^¶^ TB score: assessment of change in clinical state in patients with TB; * Not specified if VD_2_ (ergocalciferol) or VD_3_; ^±^ Authors did not assess VD levels; ^&^ Sputum smear conversion (secondary outcome): not different between groups.
